# Decoding semi-automated title-abstract screening: findings from a convenience sample of reviews

**DOI:** 10.1186/s13643-020-01528-x

**Published:** 2020-11-27

**Authors:** Allison Gates, Michelle Gates, Daniel DaRosa, Sarah A. Elliott, Jennifer Pillay, Sholeh Rahman, Ben Vandermeer, Lisa Hartling

**Affiliations:** Alberta Research Centre for Health Evidence and the University of Alberta Evidence-based Practice Center, Department of Pediatrics, University of Alberta, Edmonton, Alberta Canada

**Keywords:** Machine learning, Artificial intelligence, Text mining, Systematic reviews, Methods, Efficiency

## Abstract

**Background:**

We evaluated the benefits and risks of using the Abstrackr machine learning (ML) tool to semi-automate title-abstract screening and explored whether Abstrackr’s predictions varied by review or study-level characteristics.

**Methods:**

For a convenience sample of 16 reviews for which adequate data were available to address our objectives (11 systematic reviews and 5 rapid reviews), we screened a 200-record training set in Abstrackr and downloaded the relevance (relevant or irrelevant) of the remaining records, as predicted by the tool. We retrospectively simulated the liberal-accelerated screening approach. We estimated the time savings and proportion missed compared with dual independent screening. For reviews with pairwise meta-analyses, we evaluated changes to the pooled effects after removing the missed studies. We explored whether the tool’s predictions varied by review and study-level characteristics.

**Results:**

Using the ML-assisted liberal-accelerated approach, we wrongly excluded 0 to 3 (0 to 14%) records that were included in the final reports, but saved a median (IQR) 26 (9, 42) h of screening time. One missed study was included in eight pairwise meta-analyses in one systematic review. The pooled effect for just one of those meta-analyses changed considerably (from MD (95% CI) − 1.53 (− 2.92, − 0.15) to − 1.17 (− 2.70, 0.36)). Of 802 records in the final reports, 87% were correctly predicted as relevant. The correctness of the predictions did not differ by review (systematic or rapid, *P* = 0.37) or intervention type (simple or complex, *P* = 0.47). The predictions were more often correct in reviews with multiple (89%) vs. single (83%) research questions (*P* = 0.01), or that included only trials (95%) vs. multiple designs (86%) (*P* = 0.003). At the study level, trials (91%), mixed methods (100%), and qualitative (93%) studies were more often correctly predicted as relevant compared with observational studies (79%) or reviews (83%) (*P* = 0.0006). Studies at high or unclear (88%) vs. low risk of bias (80%) (*P* = 0.039), and those published more recently (mean (SD) 2008 (7) vs. 2006 (10), *P* = 0.02) were more often correctly predicted as relevant.

**Conclusion:**

Our screening approach saved time and may be suitable in conditions where the limited risk of missing relevant records is acceptable. Several of our findings are paradoxical and require further study to fully understand the tasks to which ML-assisted screening is best suited. The findings should be interpreted in light of the fact that the protocol was prepared for the funder, but not published a priori. Because we used a convenience sample, the findings may be prone to selection bias. The results may not be generalizable to other samples of reviews, ML tools, or screening approaches. The small number of missed studies across reviews with pairwise meta-analyses hindered strong conclusions about the effect of missed studies on the results and conclusions of systematic reviews.

## Background

Systematic reviews are foundational to evidence-informed decision-making, but are notoriously time- and resource-intensive to produce [1, 2]. Novel methods are needed if systematic review production is to keep pace with the publication of new evidence from trials, and if existing systematic reviews are to be kept up-to-date [2–4]. Title-abstract screening is one of the more time-consuming steps in the production of a systematic review [5]. Often, two independent reviewers will screen all potentially eligible records to identify the (relatively) few that are relevant. Machine learning (ML) tools offer the potential to semi-automate title-abstract screening in systematic reviews by predicting and prioritizing relevant records [6, 7]. Based on a review of studies on applications of ML for title-abstract screening, O’Mara-Eves et al. concluded that ML tools could be used safely to prioritize relevant records, and cautiously to replace one of two human reviewers [8].

In spite of the clear need to create efficiencies in systematic review production [1, 2] and the accrual of evidence highlighting the benefits and risks [8, 9], and usability [10] of available ML tools, the adoption of ML-assisted methods has been slow [8, 11, 12]. In a 2019 commentary, O’Connor et al. summarized possible barriers to adoption, including distrust by review teams and end users of systematic reviews; set-up challenges and incompatibility with traditional workflows; and inadequate awareness of available tools [13]. Most importantly, for widespread adoption to be achieved, review teams and other stakeholders need to feel confident that the application of ML-assisted title-abstract screening does not compromise the validity of the review (i.e., that important studies that could impact the results and conclusions are not erroneously omitted) [14].

Previously published studies undertaken at our evidence synthesis centre [10, 15, 16] have addressed the benefits (workload and estimated time savings) and risks (omitting relevant studies) of various ML-assisted screening approaches in systematic and rapid reviews. We have also explored the usability of some available ML tools [10]. Despite promising findings, in the absence of clear guidance or endorsement by evidence synthesis organizations, it remains unclear how ML-assisted methods should (or could) be incorporated into practice [13]. There is also little research documenting under which conditions ML-assisted screening approaches may be most successfully applied. To what extent ML-assisted methods could compromise the validity of systematic reviews’ findings is vitally important, but few studies have reported on this outcome [17, 18]. In this study, we aimed to address these knowledge gaps. For a sample of 16 reviews, we:

1. Evaluated the benefits (workload savings, estimated time savings) and risks (proportion missed) of using a ML tool’s predictions in the context of the liberal-accelerated approach to screening [19, 20] in systematic reviews and assessed the impact of missed studies on the results of the systematic reviews that included pairwise meta-analyses; and
2. Explored whether there were differences in the studies correctly predicted to be relevant by the ML tool and those incorrectly predicted to be irrelevant based on review, study, and publication characteristics.

## Methods

### Study conduct

We undertook this study in accordance to a protocol, available at 10.7939/DVN/S0UTUF (uploaded post hoc). We have reported the study in adherence to recommended standards [21].

### Rationale

Between 13 and 15 August 2019, our research librarian conducted scoping searches to assess the body of research on the use of ML tools for expediting screening in systematic reviews (Additional file 1). We also reviewed the systematic review of research investigating text mining for study identification in systematic reviews published by O’Mara-Eves et al. in 2015 [8]. We identified nine studies that were conducted or published since 2015 reporting on the use of ML for screening [10, 15, 16, 18, 22–26]. As none of the studies shared our objectives, and trustworthiness remains a serious barrier to the update of semi-automated screening by review teams [13], we saw value in undertaking the present study.

### Sample of reviews

The senior research staff (AG, MG, SAE, JP, and LH) selected a convenience sample of 16 reviews either completed or underway at our center. We selected the reviews based on the availability of adequate screening and/or study characteristics data to contribute to our objectives, in a format efficiently amenable to analysis. Table 1 shows the review-level characteristics for each, including the review type (systematic or rapid), research question type (single or multiple), intervention or exposure (simple vs. complex), and included study designs (single vs. multiple). We considered complex interventions to be those that could include multiple components as opposed to a single treatment (e.g., drug, diagnostic test); typically, these were behavioral interventions. Of the reviews, 11 (69%) were systematic reviews, 10 (63%) investigated a single research question, nine (56%) investigated simple interventions or exposures, and four (25%) included only single study designs. The sources searched for each review are in Additional file 2. All systematic reviews used comprehensive searches of electronic databases and grey literature sources, supplemented with reference list scanning. In the rapid reviews, only electronic databases were searched.

**Table 1:** Characteristics of the included reviews

Review name	Review type	Research question type	Intervention/exposure type	Included study designs
**Activity and pregnancy**	Systematic	**Single:** harms	**Complex:** occupational activity	**Multiple:** any study design
**Antipsychotics**	Systematic	**Multiple:** effectiveness and harms	**Simple:** any Food and Drug Administration-approved first- or second-generation antipsychotic	**Multiple:** RCTs and nRCTs, controlled cohort, controlled before-after
**Brain injury**	Systematic	**Multiple:** demographics, pathophysiology, predictive value, ideal follow-up time	**Simple:** brain imaging modalities	**Multiple:** RCTs, cohort, case-control
**Community gardening**	Rapid	**Single:** effectiveness	**Complex:** community garden or allotment garden	**Multiple:** any study design
**Concussion**	Systematic	**Single:** association	**Simple:** perceptions of concussions	**Multiple:** cross-sectional, cohort, mixed methods, qualitative
**Depression safety**	Rapid	**Single:** harms	**Complex:** non-pharmacologic interventions for depression	**Multiple:** RCTs, systematic reviews
**Depression treatments**	Rapid	**Single:** effectiveness	**Complex:** non-pharmacologic interventions for depression	**Single:** systematic reviews
**Diabetes**	Systematic	**Multiple:** effectiveness, harms, factors contributing to effectiveness	**Complex:** behavioral programs for type 1 and type 2 diabetes	**Multiple:** RCTs, nRCTs, prospective cohort, controlled before-after
**Digital technologies for pain**	Systematic	**Single:** effectiveness	**Simple:** any digital distractor	**Multiple:** observational, RCTs, nRCTs
**Experiences of bronchiolitis**	Systematic	**Single:** exploratory	**Simple:** experiences and information needs	**Multiple:** observational, qualitative, mixed methods
**Experiences of UTIs**	Systematic	**Single:** exploratory	**Simple:** experiences and information needs	**Multiple:** observational, qualitative, mixed methods
**Preterm delivery**	Rapid	**Multiple:** effectiveness, diagnostic accuracy	**Simple:** predictive tests for preterm delivery	**Multiple**: systematic reviews, cohort
**Treatments for bronchiolitis**	Systematic	**Multiple:** effectiveness and harms	**Simple:** any bronchodilator, any corticosteroid, hypertonic saline, oxygen therapy, antibiotics, heliox	**Single:** RCTs
**VBAC**	Systematic	**Single:** effectiveness	**Complex:** adjunct clinical interventions targeting trial of labor after cesarean and vaginal birth after cesarean rates	**Multiple:** any comparative design (RCTs, nRCTs, observational)
**Visual acuity**	Systematic	**Multiple:** effectiveness and harms	**Simple:** vision screening tests (alone or within multicomponent screening/assessment)	**Single:** RCTs
**Workplace stress**	Rapid	**Single:** effectiveness	**Complex:** workplace interventions to reduce stress, absenteeism, and mental health problems	**Single:** systematic reviews

*nRCT* non-randomized controlled trial, *RCT* randomized controlled trial, *UTI* urinary tract infection, *VBAC* vaginal birth after cesarean section

Although many modifications to standard systematic review methods may be applied in the completion of rapid reviews [27], for the purpose of this study we considered only the screening method. For the systematic reviews, title-abstract screening was completed by two independent reviewers who came to consensus on the studies included in the review. The review team typically included a senior reviewer (the reviewer who oversaw all aspects of the review and who had the most methodological and/or clinical expertise) and a second reviewer (who was involved in screening and often other review processes, like data extraction). For the rapid reviews, the screening was completed by one highly experienced reviewer (the senior reviewer). This approach is considered acceptable when evidence is needed for pressing policy and health system decisions [28].

### Machine learning tool: Abstrackr

We used Abstrackr (http://abstrackr.cebm.brown.edu) [29], an online ML tool for title-abstract screening, for this study. Among the many available tools, we chose Abstrackr because it is freely available and testing at our center found it to be more reliable and user friendly than other available tools [10]. Experienced reviewers at our center (*n* = 8) completed standard review tasks in Abstrackr and rated it, on average, 79/100 on the System Usability Scale [10] (a standard survey commonly used to subjectively appraise the usability of a product or service) [30]. In our analysis of qualitative comments, reviewers described the tool as easy to use and trustworthy and appreciated the simple and uncluttered user interface [10]. When used to assist the second reviewer in a pair (a semi-automated approach to screening), across three systematic reviews on average, only 1% (range, 0 to 2%) of relevant studies (i.e., those included in the final reviews) were missed [10].

To screen in Abstrackr, all records retrieved by the searches must first be uploaded to the system. Once the records are uploaded, titles and abstracts appear one at a time on the user interface and the reviewer is prompted to label each as ‘relevant’, ‘irrelevant’, or ‘borderline’. While screening, Abstrackr learns from the reviewer’s labels and other data via active learning and dual supervision [29]. In active learning, the reviewer must first screen a ‘training set’ of records from which the model learns to distinguish between those that are relevant or irrelevant based on common features (i.e., words or combinations of words) [29]. In dual supervision, the reviewers can communicate their knowledge of the review task to the model by tagging terms that are indicative of relevance or irrelevance (e.g., the term ‘trial’ may be imparted as relevant in systematic reviews that seek to include only trials) [29]. After screening a training set, the review team can view and download Abstrackr’s relevance predictions for records that have not yet been screened. The predictions are presented to reviewers in two ways: a numeric value representing the probability of relevance (0 to 1) and a binary ‘hard’ screening prediction (true or false, i.e., relevant or irrelevant).

### Data collection

#### Screening simulation

For each review, we uploaded all records retrieved by the searches to Abstrackr for screening. We used the single-reviewer and random citation order settings and screened a 200-record training set for each review by retrospectively replicating the senior reviewer’s original screening decisions. Abstrackr’s ability to learn and accurately predict the relevance of candidate records depends on the correct identification and labeling of relevant and irrelevant records in the training set. Replicating the senior reviewer’s decisions optimized the probability of a good quality training set. Although the optimal training set size is not known [7], the developers of a similar tool recommend a training set that includes at least 40 excluded and 10 included records, up to a maximum of 300 records [31].

For systematic reviews completed at our center, any record marked as ‘include’ (i.e., relevant) or ‘unsure’ (i.e., borderline) by either of two independent reviewers at the title-abstract screening stage is eligible for scrutiny by full text. For this reason, our screening files typically include one of two screening decisions per record: ‘include/unsure’ (relevant) or ‘exclude’ (irrelevant). Because we could not ascertain retrospectively whether the decision for each record was ‘include’ or ‘unsure’, we entered all ‘include/unsure’ decisions as ‘relevant’ in Abstrackr. We did not use the ‘borderline’ decision.

After screening the training set, we downloaded the predicted relevance of the remaining records. Typically, these became available within 24 h. In instances where the predictions did not become available in 48 h, we continued to screen in batches of 100 records until they did. We used the hard screening predictions instead of applying custom thresholds based on the relevance probabilities for each remaining record. In the absence of guidance on the optimal threshold to apply, using the hard screening predictions was likely realistic of how the tool is used by review teams.

Although potentially prone to bias, the liberal-accelerated screening approach [19, 20] saves time in traditional systematic reviews even without the use of ML. In this approach, any record marked as ‘include’ or ‘unsure’ by either of two independent reviewers automatically moves forward to full-text screening. Only records marked as ‘exclude’ by one reviewer are screened by a second reviewer to confirm or refute their exclusion. The time-consuming step of achieving consensus at the title-abstract level becomes irrelevant and is omitted.

Building on earlier findings from a similar sample of reviews [15], we devised a retrospective screening simulation to investigate the benefits and risks of using ML in combination with the liberal-accelerated screening approach, compared with traditional dual independent screening. In this simulation, after screening a training set of 200 records, the senior reviewer would download the predictions and continue screening only those that were predicted to be relevant. The second reviewer would screen only the records excluded either by the senior reviewer or predicted to be irrelevant by Abstrackr to confirm or refute their exclusion. This simulation was relevant only to the systematic reviews, for which dual independent screening had been undertaken. Since a single reviewer completed study selection for the rapid reviews, retrospectively simulating liberal-accelerated screening for these reviews was not possible.

#### Differences in review results

To investigate differences in the results of systematic reviews when relevant studies are omitted, for systematic reviews with pairwise meta-analyses we re-ran the analyses for the primary outcomes of effectiveness omitting the studies that would have been erroneously excluded from the final reports via the semi-automated liberal-accelerated simulation. We investigated differences in the results only of systematic reviews with pairwise meta-analyses because the appraisal of this outcome among reviews with qualitative or quantitative narrative syntheses was not feasible within available time and resources. When the primary outcomes were not explicitly reported, we considered any outcome for which certainty of evidence appraisals were reported to be primary outcomes. Otherwise, we considered the first reported outcome to be the primary outcome.

#### Characteristics of missed studies

We pooled the data for the studies included in the final reports for all reviews to explore which characteristics might be associated with correctly or incorrectly labeling relevant studies. From the final report for each review, we extracted the risk of bias (low, unclear, or high) and design (trial, observational, mixed methods, qualitative, or review) of each included study. For reviews that included study designs other than randomized trials, we considered methodological quality as inverse to risk of bias. We categorized the risk of bias based on the retrospective quality scores derived from various appraisal tools (Additional file 3). We also documented the year of publication and the impact factor of the journal in which each included study was published based on 2018 data reported on the Journal Citation Reports website (Clarivate Analytics, Philadelphia, Pennsylvania). A second investigator verified all extracted data prior to analysis.

### Data analysis

We exported the data to SPSS Statistics (v.25, IBM Corporation, Armonk, New York) or StatXact (v.12, Cytel Inc., Cambridge, Massachusetts) for analysis. To evaluate the benefits and risks of using Abstrackr’s predictions in the context of liberal-accelerated screening in systematic reviews, we used data from 2 × 2 cross-tabulations to calculate standard metrics [8], as follows:

- Proportion missed (error): of the studies included in the final report, the proportion erroneously excluded during title and abstract screening.
- Workload savings (absolute screening reduction): of the records that need to be screened at the title and abstract stage, the proportion that would not need to be screened manually.
- Estimated time savings: the estimated time saved by not screening records manually. We assumed a screening rate of 0.5 min per record and an 8-h work day [32].

To determine the effect of missed studies on the results of systematic reviews with pairwise meta-analyses, we compared the pooled effect estimate, 95% confidence interval, and statistical significance when missed studies were removed from the meta-analyses to those from the original review. We did not appraise changes in clinical significance.

To explore which review, study, and publication characteristics might affect the correctness of Abstrackr’s predictions, we first compared the proportion of studies incorrectly predicted as irrelevant by Abstrackr by review type (i.e., inclusion of only trials vs. multiple study designs; single vs. multiple research questions; systematic review vs. rapid review; complex vs. simple interventions) and by study characteristics (study design (trial, observational, mixed methods, qualitative, review) and risk of bias (low or unclear/high)) via Fischer exact tests. We compared the mean (SD) year of publication and impact factor of the journals in which studies were published among those that were correctly and incorrectly labeled via unpaired *t* tests.

## Results

Screening characteristics (Table 2) for the included reviews have been reported in a separate study investigating additional unique simulations [15]. The screening workload (retrospective) varied by review (median (IQR [25th percentile, 75th percentile]), 2123 (1321, 5961) records). The workload tended to be larger for the systematic reviews (5092 (2078, 8746) records) compared to the rapid reviews (964 (767, 1536) records). Across reviews, a median (IQR) 9 (4, 14)% candidate records were included following title and abstract screening (8 (3, 9)% for the systematic reviews and 18 (9, 21)% for the rapid reviews). A median (IQR) 2 (0.4, 4)% candidate records were included in the final reports (0.6 (0.4, 2)% in the systematic reviews and 8 (2, 8)% in the rapid reviews). After screening the training sets, across reviews Abstrackr predicted that a median (IQR) 32 (13, 41)% of those remaining were relevant (25 (12, 34)% for the systematic reviews and 38 (37, 59)% for the rapid reviews).

**Table 2:** Screening characteristics of the included reviews

Review	Screened by human reviewers, ***n*** (%)^a^			Screened in Abstrackr, ***n*** (%)	
	Screening workload	Included, title and abstract	Included, final report	Training set (***n*** includes/excludes, % includes)^**b**^	Predicted relevant
**Systematic reviews**					
Activity and pregnancy	2928	236 (8)	98 (3)	10/190 (5)	319 (12)
Antipsychotics	12156	1177 (10)	127 (1)	15/185 (8)	2117 (18)
Brain injury	6262	518 (8)	40 (1)	11/189 (6)	2126 (35)
Concussion	1439	46 (3)	5 (< 1)	3/197 (2)	638 (51)
Diabetes	47141	698 (1)	205 (< 1)	104/196 (52)	5187 (11)
Digital technologies for pain	2662	207 (8)	64 (2)	15/185 (8)	321 (13)
Experiences of bronchiolitis	651	88 (14)	28 (4)	13/187 (7)	111 (25)
Experiences of UTIs	1493	25 (2)	4 (< 1)	3/197 (2)	864 (67)
Treatments for bronchiolitis	5861	518 (9)	137 (2)	12/188 (6)	656 (12)
VBAC	5092	807 (16)	21 (< 1)	25/175 (13)	1490 (30)
Visual acuity	11229	224 (2)	1 (< 1)	4/296 (1)	3639 (33)
**Rapid reviews**					
Community gardening	1536	153 (10)	32 (2)	55/145 (28)	139 (10)
Depression safety	964	44 (5)	8 (1)	7/193 (4)	449 (59)
Depression treatments	1583	418 (26)	179 (11)	43/157 (22)	904 (65)
Preterm delivery	451	96 (21)	34 (8)	47/153 (24)	95 (38)
Workplace stress	767	141 (18)	59 (8)	36/164 (18)	210 (37)

*UTI* urinary tract infection, *VBAC* vaginal birth after cesarean

^a^Retrospective screening data

^b^The training set was 200 records for all reviews except diabetes and visual acuity, for which it was 300

### Liberal-accelerated screening simulation

Table 3 shows the proportion missed, workload savings, and estimated time savings had the reviewers leveraged Abstrackr’s predictions and the liberal-accelerated screening approach in each systematic review. Records missed are those that are included in the final report, but were excluded via the simulated approach at the title-abstract screening stage. To ascertain whether the simulated approach provided any advantage over screening by a single reviewer, we have also included a column showing the number and proportion of studies that the second reviewer would have missed had they screened the records in isolation.

**Table 3:** Proportion missed, workload savings, and estimated time savings for each systematic review

Systematic review	Records missed, single reviewer, ***n*** (%)	Records missed, simulation, ***n*** (%)	Workload savings, ***n*** (%)	Estimated time savings, h (d)
**Activity and pregnancy**	11 (11)	1 (1)	2536 (43)	21 h (3 d)
**Antipsychotics**	4 (3)	3 (2)	10508 (43)	88 h (11 d)
**Brain injury**	2 (5)	0 (0)	4193 (33)	35 h (4 d)
**Concussion**	0 (0)	0 (0)	635 (22)	5 h (< 1 d)
**Digital technologies for pain**	0 (0)	0 (0)	2271 (43)	19 h (2 d)
**Experiences of bronchiolitis**	12 (43)	3 (11)	389 (30)	3 h (< 1 d)
**Experiences of UTIs**	0 (0)	0 (0)	448 (15)	4 h (< 1 d)
**Treatments for bronchiolitis**	10 (7)	1 (1)	5300 (45)	44 h (6 d)
**VBAC**	5 (24)	3 (14)	3750 (37)	31 h (4 d)
**Visual acuity**	0 (0)	0 (0)	7418 (33)	62 h (8 d)

*d* days, *h* hours, *UTI* urinary tract infection, *VBAC* vaginal birth after cesarean

The diabetes review was excluded because the screening data were not in a format amenable to analysis

Compared to dual independent screening, for five (50%) of the systematic reviews no studies were erroneously excluded via our simulated approach. In two (20%) systematic reviews, one record was erroneously excluded, equivalent to 1% of the included records in both reviews. In the remaining three (30%) reviews, three records were erroneously excluded, equivalent to 2 to 14% of the included studies. The simulated approach was advantageous (i.e., fewer records were missed) relative to screening by a single reviewer in six (60%) of the systematic reviews; in many cases, the difference was substantial (e.g., 11% vs. 43% in the Experiences of bronchiolitis review; 1% vs. 11% in the Activity and pregnancy review; 1% vs. 7% in the Treatments for bronchiolitis review; 14% vs. 24% for the VBAC review; 0% vs. 5% in the Brain injury review).

The median (IQR) workload savings across reviews was 3143 (1044, 5023) records (35 (30, 43) %) compared to dual independent screening. This equated to a median (IQR) estimated time savings of 26 (9, 42) h or 3 (1, 5) working days of uninterrupted screening.

### Impact of missed studies on the results

Among the five systematic reviews where studies were missed, three included pairwise meta-analyses (Activity and pregnancy, Antipsychotics, and Treatment for bronchiolitis) (Additional file 4). The single missed study for each of the Activity and pregnancy and Treatments for bronchiolitis reviews were not included in any of the meta-analyses. It is notable that the missed study in the Activity and pregnancy review was written in Chinese, although it did include an English abstract. Neither of the studies reported on the primary outcomes of their respective systematic reviews.

For Antipsychotics, there were three missed studies. Of the 49 pairwise comparisons for which there was at least low strength of evidence in the final report, one of the missed studies (McCracken et al., 2002) was included in 8 (16%) comparisons. The 8 meta-analyses compared second-generation antipsychotics (SGAs) to placebo for the following outcomes for autism spectrum disorder: irritability, lethargy/social withdrawal, stereotypy, inappropriate speech, compulsions, response rate, discontinuation due to lack of efficacy, and appetite increase. Additional file 5 shows the pooled estimate of effect (95% CI) and statistical significance for the 8 relevant meta-analyses in the original report and following the removal of the study by McCracken et al. The statistical significance of the pooled estimate of effect changed in one of the meta-analyses (i.e., 2% of all comparisons for which there was at least low strength of evidence included in the report). For children with autism spectrum disorder, the original meta-analysis found a statistically significant reduction in compulsions in favor of SGAs (mean difference (MD) (95% CI), − 1.53 (− 2.92, − 0.15), *P* = 0.03). The effect was no longer statistically significant following the removal of McCracken et al. from the analysis (MD (95% CI), − 1.17 (− 2.70, 0.36), *P* = 014). Otherwise, removing McCracken et al. from relevant meta-analyses did not result in changes in point estimates or confidence intervals that impacted the statistical significance of the findings.

Although not included in any of the meta-analyses, the large retrospective cohort study by Bobo et al. (2013) contributed low certainty evidence of an increased risk for type 2 diabetes among patients taking SGAs. No other studies contributed data for this outcome. Although the prospective study by Correll et al. (2009) contributed to the network meta-analysis for harms, it did not report on any of the intermediate or effectiveness outcomes.

### Association of study, review, and publication characteristics with predictions

The pooled dataset for the studies included in the 16 final reports contained 802 records for which Abstrackr had made a prediction (excludes those included in the training sets). Among these, Abstrackr correctly predicted that 696 (87%) were relevant and incorrectly predicted that 106 (13%) were irrelevant after the 200-record training set.

### Review characteristics

Table 4 shows the characteristics of the reviews, stratified by the correctness of Abstrackr’s relevance predictions. Six hundred eighty-nine (86%) studies were included across the systematic reviews and 113 (14%) across the rapid reviews. There was no difference (*P* = 0.37) in Abstrackr’s ability to correctly predict the relevance of studies by review type (*n* = 601 (87%) of studies in systematic reviews and 95 (84%) of those in rapid reviews were correctly identified).

**Table 4:** Select review characteristics, stratified by Abstrackr’s relevance predictions

Review characteristic	***n*** studies	Correctly predicted as relevant, ***n*** (%)	Incorrectly predicted as irrelevant, ***n*** (%)	***P*** value^a^
**Review type**				
Systematic	689	601 (87)	88 (13)	0.37
Rapid	113	95 (84)	18 (16)	
**Research question**				
Single	297	246 (83)	51 (17)	0.01
Multiple	505	450 (89)	55 (11)	
**Intervention/exposure**				
Simple	403	346 (86)	57 (14)	0.47
Complex	399	350 (88)	49 (12)	
**Included study designs**				
Single—only randomized trials	129	122 (95)	7 (5)	0.003
Single—only systematic reviews	72	57 (79)	15 (21)	
Multiple	601	517 (86)	84 (14)	

^a^Fisher’s exact test

Two hundred ninety-seven (37%) studies were included in reviews that answered a single research question, and 505 (63%) were included in reviews that answered multiple questions. There was a statistically significant difference (*P* = 0.01) in Abstrackr’s ability to correctly predict the relevance of studies by research question type. Four hundred fifty (89%) studies in reviews with multiple research questions were correctly identified. The proportion of correctly identified studies was less (*n* = 246, 83%) in reviews with a single research question.

Four hundred three (50%) studies were included in reviews that tested a simple intervention/exposure, and 399 (50%) were included in reviews that tested complex interventions. There was no difference (*P* = 0.47) in Abstrackr’s ability to correctly predict the relevance of studies by intervention- or exposure-type (*n* = 346 (86%) studies in reviews of simple interventions and 350 (88%) studies in reviews of complex interventions were correctly identified).

Two hundred one (25%) studies were included in reviews that included only one study design (trials or systematic reviews), while the remaining 601 (75%) were included in reviews that included multiple designs (including observational studies). There was a statistically significant difference (*P* = 0.003) in Abstrackr’s ability to correctly predict the relevance of studies by included study designs. Abstrackr correctly predicted the relevance of 122 (95%) studies in reviews that included only randomized trials as compared to 57 (79%) and 517 (86%) in reviews that included only systematic reviews, or multiple study designs, respectively.

### Study characteristics

Table 5 shows the characteristics of the studies, stratified by Abstrackr’s relevance predictions. Of the included studies, 483 (60%) were trials, 214 (27%) were observational, 2 (0.2%) were mixed methods, 15 (2%) were qualitative, and 88 (11%) were reviews. There was a statistically significant difference (*P* = 0.0006) in Abstrackr’s ability to correctly predict the relevance of included studies by study design. Abstrackr correctly predicted the relevance of 438 (91%) of the trials, 2 (100%) of the mixed methods studies, and 14 (93%) of the qualitative studies. By comparison, the proportion of correct predictions was less for observational studies (*n* = 214, 79%) and reviews (*n* = 88, 83%).

**Table 5:** Study design and study-level risk of bias, stratified by Abstrackr’s relevance predictions

Study characteristic	***N*** studies	Correctly predicted as relevant, ***n*** (%)	Incorrectly predicted as irrelevant, ***n*** (%)	***P*** value^a^
**Design**				
Trial	483	438 (91)	45 (9)	0.0006
Observational	214	169 (79)	45 (21)	
Mixed methods	2	2 (100)	0 (0)	
Qualitative	15	14 (93)	1 (7)	
Review	88	73 (83)	15 (17)	
**Risk of bias**				
Low	120	96 (80)	24 (20)	0.039
High or unclear	500	438 (88)	62 (12)	

^a^Fisher’s exact test

Of the 620 studies for which we had risk of bias details, 120 (19%) were at low and 500 (81%) were at unclear or high overall risk of bias. There was a statistically significant difference (*P* = 0.039) in Abstrackr’s ability to correctly predict the relevance of included studies by risk of bias. Abstrackr correctly predicted the relevance of 438 (88%) of studies at unclear or high risk of bias as compared to 96 (80%) of those at low risk of bias.

### Publication characteristics

Table 6 shows the characteristics of the publications, stratified by Abstrackr’s relevance predictions. Across all studies, the mean (SD) publication year was 2008 (7). There was a statistically significant difference (*P* = 0.02) in Abstrackr’s ability to correctly identify relevant studies by publication year. The mean (SD) year of publication was 2008 (7) for studies correctly identified compared to 2006 (10) for those erroneously excluded (mean difference (95% CI), 1.77 (0.27, 3.26). This difference is not considered practically significant.

**Table 6:** Publication year and journal impact factor, stratified by Abstrackr’s relevance predictions

Study characteristic	All studies	Correctly predicted as relevant	Incorrectly predicted as irrelevant	Mean difference (95% CI)^a^	***P*** value^b^
**Publication year, mean (SD)**	2008 (7)	2008 (7)	2006 (10)	1.77 (0.27, 3.26)	0.02
**Impact factor, mean (SD)**	4.87 (8.49)	4.91 (8.39)	4.61 (9.14)	0.30 (− 1.44, 2.03)	0.74

^a^Mean difference between correctly identified studies and those erroneously excluded

^b^Unpaired *t* test

The mean (SD) impact factor for the journals in which the studies were published was 4.87 (8.49). There was no difference (*P* = 0.74) in Abstrackr’s ability to correctly identify relevant studies by the impact factor for the journal in which they were published. The mean (SD) impact factor was 4.91 (8.39) for studies correctly identified as relevant and 4.61 (9.14) for those erroneously excluded (mean difference (95% CI), 0.30 (− 1.44, 2.03)).

## Discussion

Compared with dual independent screening, leveraging Abstrackr’s predictions in combination with a liberal-accelerated screening approach resulted in few, if any, missed records (between 0 and 3 records per review, or 0 to 14% of those included in the final reports). The missed records would not have changed the conclusions for the main effectiveness outcomes in the impacted reviews; moreover, as we have previously shown it is likely that in the context of a comprehensive search, missed studies would be identified by other means (e.g., reference list scans) [15]. The workload savings were substantial, and despite being not quite as efficient, considerably fewer studies were missed compared to screening by a single reviewer in many (60%) reviews. Included studies were correctly identified more frequently among reviews that included multiple research questions (vs. single) and those that included only randomized trials (vs. only reviews, or multiple study designs). Correctly identified studies were more likely to be randomized trials, mixed methods, and qualitative studies (vs. observational studies and systematic reviews).

As part of our previous work, we simulated four additional methods whereby we could leverage Abstrackr’s predictions to expedite screening, including fully automated and semi-automated approaches [15]. The simulation that provided the best balance of reliability and workload savings was a semi-automated second screener approach, based on an algorithm first reported by Wallace et al. in 2010 [32]. In this approach, the senior reviewer would screen a 200-record training set and continue to screen only those that Abstrackr predicted to be relevant. The second reviewer would screen all records as per usual. The second reviewer’s decisions and those of the senior reviewer and Abstrackr would be compared to determine which would be eligible for scrutiny by full text. Among the same sample of reviews, the records that were missed were identical to those in the liberal-accelerated simulation. The median (IQR) workload savings was 2409 (3616) records, equivalent to an estimated time savings of 20 (31) h or 3 (4) working days. Thus, compared to the semi-automated second screener approach [32], the liberal-accelerated approach resulted in marginally greater workload and time savings without compromising reliability.

In exploring the screening tasks for which ML-assisted screening might be best suited, some of our findings were paradoxical. For example, studies were more often correctly identified as relevant in systematic reviews with multiple research questions (vs. a single question). There was no difference in the proportion of studies correctly identified as relevant among systematic reviews that investigated complex vs. simple interventions. There are likely a multitude of interacting factors that affect Abstrackr’s predictions, including the size and composition of the training sets. More research is needed to inform a framework to assist review teams in deciding when or when not to use ML-assisted methods. Our findings are consistent with other studies which have suggested that ML may be particularly useful for expediting simpler review tasks (e.g., differentiating trials from studies of other designs) [33], leaving more complex decisions to human experts. Cochrane’s RCT classifier, which essentially automates the identification of trials, is one example of such an approach [33]. By automatically excluding ‘obviously irrelevant’ studies, human reviewers are left to screen only those where screening decisions are more ambiguous.

Our data suggest that combining Abstrackr’s early predictions with the liberal-accelerated screening method may be an acceptable approach in reviews where the limited risk of missing a small number of records is acceptable (e.g., some rapid reviews), or the outcomes are not critical. This may be true for some scoping reviews, where the general purpose is to identify and map the available evidence [34], rather than synthesize data on the effect of an intervention on one or more outcomes. Even for systematic reviews, the recently updated Cochrane Handbook states that the selection of studies should be undertaken by two reviewers working independently, but that a single reviewer is acceptable for title-abstract screening [35]. Similarly, the AMSTAR 2 tool, used to judge the confidence in the results of systematic reviews [36], states that title-abstract screening by a single reviewer is acceptable if good agreement (at least 80%) between two reviewers was reached during pilot testing. The ML-assisted approach that we have proposed provides a good compromise between dual independent screening (most rigorous) and single-reviewer screening (less rigorous) for review teams looking to save time and resources while maintaining or exceeding the methodological rigour expected for high-quality systematic reviews [35, 36].

When conceptualizing the relative advantages of semi-automatic title-abstract screening, it will be important to look beyond study selection to other tasks that may benefit from the associated gains in efficiency. For example, published systematic reviews frequently report limits to the searches (e.g., limited databases, published literature only) and eligibility criteria (e.g., trials only, English language only) [37], both of which can have implications for the conclusions of the review. If studies can be selected more efficiently, review teams may choose to broaden their searches or eligibility criteria, potentially missing *fewer* studies even if a small proportion are erroneously omitted through semi-automation.

Given the retrospective nature of most studies, the semi-automation of different review tasks have largely been studied as isolated processes. Prospective studies are needed to bridge the gap between hypothetical opportunities and concrete demonstrations of the risks and benefits of various novel approaches. For example, recently a full systematic review was completed in two weeks by a research team in Australia using a series of semi-automated and manual processes [38]. The authors reported on the facilitators and barriers to their approaches [38]. To build trust, beyond replication of existing studies it will be important for review teams to be able to conceptualize, step-by-step, how ML can be integrated into their standard procedures [13] and under what circumstances the benefits of different approaches outweigh the inherent risks. As a starting point, prospective direct comparisons of systematic reviews completed with and without ML-assisted methods would be helpful to encourage adoption. There may be ways to incorporate such evaluations into traditional systematic review processes without substantially increasing reviewer burden.

## Strengths and limitations

This is one of few studies to report on the potential impact of ML-assisted title-abstract screening on the results and conclusions of systematic reviews, and to explore the correctness of predictions by review, study, and publication-level characteristics. The findings should be interpreted in light of the fact that the protocol was approved by our funder, but not published a priori. Our findings are potentially prone to selection bias, as we evaluated a convenience sample of reviews for which adequate data were available for analysis. Although many tools and methods are available to semi-automate title-abstract screening, we used only Abstrackr and simulated a liberal-accelerated approach. The findings should not be generalized to other tools or approaches, or other samples of reviews. Moreover, we used relatively small training sets in an attempt to maximize efficiency. It is possible that different training sets would have yielded more or less favorable results. The retrospective screening results for the rapid reviews are more prone to error and bias compared with those for the systematic reviews because a single reviewer completed study selection. Since a machine learning tool’s predictions can only be as good as the data on which it was trained, it is possible that the study selection method used for the rapid reviews negatively impacted the accuracy of the predictions. Our findings related to the changes to a review’s conclusions are applicable only to the reviews with pairwise meta-analyses, of which there were few. Further, because so few studies were missed, we were only able to assess for changes to eight meta-analyses in one systematic review. The findings should be interpreted cautiously and not extrapolated to reviews with other types of syntheses (e.g., narrative). Because our evaluation was retrospective, we estimated time savings based on a screening rate of two records per minute. Although ambitious, this rate allowed for more conservative estimates of time savings and for comparisons to previous studies that have used the same rate [10, 15, 16, 39].

## Conclusions

Our ML-assisted screening approach saved considerable time and may be suitable in contexts where the limited risk of missing relevant records is acceptable (e.g., some rapid reviews). ML-assisted screening may be most trustworthy for reviews that seek to include only trials; however, as several of our findings are paradoxical, further study is needed to understand the contexts in which ML-assisted screening is best suited. Prospective evaluations will be important to fully understand the implications of adopting ML-assisted systematic review methods, build confidence among systematic reviewers, and to gather reliable estimates of time and resource savings.

## Supplementary Information

**Additional file 1.** Scoping searches. Details of the scoping searches.

**Additional file 2.** Sources searched for each review. This table shows the sources searched for each review included in this study.

**Additional file 3.** Risk of bias categorization by quality score. This table shows how we categorized risk of bias for studies that measured methodological quality via a variety of different tools.

**Additional file 4.** Missed studies for systematic reviews with meta-analyses. This table shows the studies that were classified as irrelevant via our semi-automated screening approach, yet were included in the meta-analyses in the included systematic reviews.

**Additional file 5.** Impact of the missed study on the results of relevant meta-analyses in the Antipsychotics systematic review. This table shows the pooled effect estimates for relevant meta-analyses with and without the study by McCracken et al. (2002), which was incorrectly classified as irrelevant via our semi-automated screening approach.

## Data Availability

The protocol (10.7939/DVN/S0UTUF) and datasets (10.7939/DVN/8HSDX5) generated and analyzed during the current study are available from the corresponding author upon reasonable request.

## Abbreviations

CI: Confidence interval
IQR: Interquartile range
ML: Machine learning
SGA: Second-generation antipsychotic
