# Supplementary material for: Decoding semi-automated title-abstract screening: findings from a convenience sample of reviews
Source: Syst Rev. 2020 Nov 27;9:272. doi: 10.1186/s13643-020-01528-x (PMC7694314; doi:10.1186/s13643-020-01528-x)
Supplement: Supplementary file 1 — Additional file 1. Scoping searches. Details of the scoping searches. [file 13643_2020_1528_MOESM1_ESM.docx]

**Additional file 1.** Scoping searches

**REFERENCE TRACKING – MACHINE LEARNING ARTICLES**

**Start Date:** 13-Aug-2019

**Procedure*:** For each target article, we searched for "citing" references (Google Scholar, Scopus), "cited" references (reference lists), created Medline and Google Scholar searches, and ran a "similar articles" search (PubMed).

**Target Articles (n = 8):**

**SUMMARY OF ALL DATABASE SEARCHES**

| **Database** | **Date of search** | **Results with duplicates** | **Results without duplicates** |
| --- | --- | --- | --- |
| Medline Related References | 13 Aug 2019 | 567 | 562 |
| Scopus Citing Articles | 13 Aug 2019 | 382 | 350 |
| Scopus Cited Articles (reference) | 13 Aug 2019 | 125 | 99 |
| Google Scholar Citing Articles | 14 Aug 2019 | 580 | 123 |
| Google Scholar Search String* | 14 Aug 2019 | 200 | 142 |
| Pubmed Similar Articles | 14 Aug 2019 | 514 | 337 |
| **TOTAL** | | **2368** | **1613** |

*searched first 20 pages of results

**SEARCH DETAILS**

**1. MEDLINE search for articles**

On 13 August 2019, DKL ran a federated search in Medline using the same MeSH terms and keywords found in the target articles (resulted in 567 records).

| **#** | **Searches** | **Results** |
| --- | --- | --- |
| 1 | exp Machine Learning/ | 12594 |
| 2 | Automation/ | 17285 |
| 3 | Data mining/ | 7562 |
| 4 | Electronic Data Processing/ | 13019 |
| 5 | Pattern Recognition, Automated/ | 24595 |
| 6 | Natural Language Processing/ | 3886 |
| 7 | Text Mining/ | 7562 |
| 8 | (automat* or machine learn*).tw,kf. | 233243 |
| 9 | Abstrackr.tw,kf. | 4 |
| 10 | Rayyan.tw,kf. | 19 |
| 11 | or/1-10 | 274695 |
| 12 | Systematic reviews as topic/ | 2433 |
| 13 | systematic review*.tw,kf,ab. | 149121 |
| 14 | "Abstracting and Indexing as Topic"/ | 4660 |
| 15 | Review Literature as Topic/ | 7456 |
| 16 | ((literature or abstract* or review*) adj2 screen*).tw,kf. | 5788 |
| 17 | or/12-16 | 160694 |
| 18 | Reproducibility of Results/ | 380090 |
| 19 | Decision Making/ | 89818 |
| 20 | Reliab*.tw,kf. | 453138 |
| 21 | Usab*.tw,kf. | 20545 |
| 22 | effective*.tw. | 1823924 |
| 23 | or/18-22 | 2595171 |
| 24 | 11 and 17 and 23 | 567 |

**2. Scopus Citing/Cited References**

- On 13 August 2019 DKL ran a Citing/Cited References search for each target article. The results were 382 for citing articles, and 125 for cited articles.

**The following target articles not indexed in Scopus, and were excluded from the search:**

None.

**3. Google Scholar Citing References**

- On 14 August 2019 DKL searched each target article in Google Scholar.

**Table 3.** Details of the Google Scholar Citing References Searches

| **Target article** | **Date searched** | **# cited references with duplicates (within database)** | **# cited references without duplicates (within database)** |
| --- | --- | --- | --- |
| 1. Cleo 2019 | 15 August 2019 | 0 |  |
| 2. Gates 2018 | 15 August 2019 | 8 |  |
| 3. Olofsson 2017 | 15 August 2019 | 9 |  |
| 4. Ouzzani 2016 | 15 August 2019 | 375 |  |
| 5. Rathbone 2015 | 15 August 2019 | 40 |  |
| 6. Van Altena 2019 | 15 August 2019 | 4 |  |
| 7. Wallace 2012 | 15 August 2019 | 144 |  |
| **TOTAL** |  | **580** | 123 |

**4. Google Scholar Search**

- On 14 August 2019 DKL ran the search shown below. The first 20 pages of results were reviewed only.

| ("machine learning" or automation) and ("review screening" or "abstract screening") |
| --- |

* The first 20 pages of results were reviewed.

**5. Pubmed (NCBI) similar articles**

- On 14 Aug 2019 DKL ran a Similar Articles search for the target articles shown below (resulted in 514 records).

| 31221212[uid] or 29530097[uid] or 28374510[uid] or 27919275[uid] or  26073974[uid] or 30561081[uid] |
| --- |

**The following target article was not indexed in Pubmed and was excluded from the search:**

1. Wallace BC, Small K, Brodley CE, Lau J, Trikalinos TA. Deploying an interactive machine learning system in an evidence-based practice center: Abstrackr. proceedings of the 2nd ACM SIGHIT International Health Informatics Symposium; 2012 ACM
