# Supplementary material for: Decoding semi-automated title-abstract screening: findings from a convenience sample of reviews
Source: Syst Rev. 2020 Nov 27;9:272. doi: 10.1186/s13643-020-01528-x (PMC7694314; doi:10.1186/s13643-020-01528-x)
Supplement: Supplementary file 2 — Additional file 2. Sources searched for each review. This table shows the sources searched for each review included in this study. [file 13643_2020_1528_MOESM2_ESM.docx]

**Additional file 2.** Sources searched for each review

| **Review name** | **Review type** | **Sources searched** |
| --- | --- | --- |
| **Activity and pregnancy** | Systematic | Electronic databases: Medline, Embase, Cochrane Library, CINAHL, Science Citation Index Expanded  Conference proceedings: Conference Proceeding Citation Index-Science  Trial registries: ClinicalTrials.gov |
| **Antipsychotics** | Systematic | Electronic databases: Medline, Embase, Cochrane Library, PsycINFO, CINAHL, TOXLINE  Dissertations and theses: Dissertations and Theses International  Conference proceedings: American Academy of Child and Adolescent Psychiatry, International College of Neuropsychopharmacology, International Society for Bipolar Disorders  Trial registries: ClinicalTrials.gov, WHO ICTRP  Unpublished studies: Drugs@FDA, drug manufacturers |
| **Brain injury** | Systematic | Electronic databases: Medline, Embase, Cochrane Library, Biosis Previews, Science Citation Index Expanded |
| **Community gardening** | Rapid | Electronic databases: Medline, PsycINFO |
| **Concussion** | Systematic | Electronic databases: Medline, Embase, CINAHL, PsycINFO, SPORTDiscus  Dissertations and theses: ProQuest Dissertations & Theses Global  Unpublished studies: Google |
| **Depression safety** | Rapid | Electronic databases: PubMed |
| **Depression treatments** | Rapid | Electronic databases: PubMed |
| **Diabetes** | Systematic | Electronic databases: Medline, Embase, Cochrane Library, PsycINFO, CINAHL, PubMed  Conference proceedings: Association of Diabetes Care & Education Specialists, American Diabetes Association, Canadian Diabetes Association, European Association for the Study of Diabetes, International Diabetes Federation, Society of Behavioral Medicine, International Society of Behavioral Nutrition and Physical Activity  Trial registries: ClinicalTrials.gov, WHO ICTRP |
| **Digital technologies for pain** | Systematic | Electronic databases: Medline, Embase, Cochrane Library, CINAHL, PsycINFO, IEEE Xplore, Ei Compendex, Web of Science, CINAHL, PsycINFO, IEEE Xplore, Ei Compendex, Web of Science  Trial registries: ClinicalTrials.gov  Hand search: CADTH, TRIP Database  Unpublished studies: Google |
| **Experiences of bronchiolitis** | Systematic | Electronic databases: Medline, PsycINFO, CINAHL  Dissertations and theses: ProQuest Dissertations and Theses Global |
| **Experiences of UTIs** | Systematic | Electronic databases: Medline, PsycINFO, CINAHL  Dissertations and theses: ProQuest Dissertations and Theses Global |
| **Preterm delivery** | Rapid | Electronic databases: PubMed via National Center for Biotechnology Information Entrez, Cochrane Library, Cochrane Database of Systematic Reviews, Database of Abstracts of Review of Effects, the Health Technology Assessment Database, the Centre for Reviews and Dissemination Database, the National Guidelines Clearinghouse, TRIP Database, |
| **Treatments for bronchiolitis** | Systematic | Electronic databases: Medline, Embase, Cochrane Library, CINAHL  Conference proceedings: Canadian Pediatric Academy, Pediatric Academy Societies, Society for Academic Emergency Medicine, European Respiratory Society, American Thoracic Society, European Society for Pediatric Research  Trial registries: ClinicalTrials.gov, WHO ICTRP |
| **VBAC** | Systematic | Electronic databases: Medline, Embase, Cochrane Library, CINAHL, PsycINFO  Dissertations and theses: ProQuest Dissertations and Theses Global  Conference proceedings: Cochrane Proceeding Citation Indexes, The Society for Maternal-Fetal Medicine, the Society of Obstetricians and Gynaecologists of Canada, the American Congress of Obstetricians and Gynecologists  Trial registries: ClinicalTrials.gov |
| **Visual acuity** | Systematic | Electronic databases: Medline, Embase, Cochrane Library, CINAHL, PubMed  Dissertations and theses: ProQuest Dissertations and Theses Global  Trial registries: ClinicalTrials.gov, WHO ICTRP |
| **Workplace stress** | Rapid | Electronic databases: Medline, PsycINFO, Business Source Complete |
