# Supplementary material for: Decoding semi-automated title-abstract screening: findings from a convenience sample of reviews
Source: Syst Rev. 2020 Nov 27;9:272. doi: 10.1186/s13643-020-01528-x (PMC7694314; doi:10.1186/s13643-020-01528-x)
Supplement: Supplementary file 3 — Additional file 3. Risk of bias categorization by quality score. This table shows how we categorized risk of bias for studies that measured methodological quality via a variety of different tools. [file 13643_2020_1528_MOESM3_ESM.docx]

**Additional file 3. Risk of bias categorization by quality score**

| **Tool** | **Highest possible score** | **Risk of bias, by quality score** | | |
| --- | --- | --- | --- | --- |
|  |  | **Low** | **Unclear** | **High** |
| AMSTAR | 11 | ≥8 | 4 – 7 | ≤3 |
| AMSTAR, adapted | 10 | ≥7 | 3 – 6 | ≤2 |
| Mixed methods appraisal tool (2008) | 4 | ≥3 | 2 | ≤1 |
| Mixed methods appraisal tool (2018) | 5 | ≥4 | 2 – 3 | ≤1 |
| Newcastle Ottawa Scale, adapted | 5 | ≥4 | 2 – 3 | ≤1 |
| NIH Quality Appraisal Tools | Good | Good | Fair | Poor |

AMSTAR = Assessing the Methodological Quality of Systematic Reviews; NIH = National Institutes of Health.
