# Supplementary material for: Decoding semi-automated title-abstract screening: findings from a convenience sample of reviews
Source: Syst Rev. 2020 Nov 27;9:272. doi: 10.1186/s13643-020-01528-x (PMC7694314; doi:10.1186/s13643-020-01528-x)
Supplement: Supplementary file 4 — Additional file 4. Missed studies for systematic reviews with meta-analyses. This table shows the studies that were classified as irrelevant via our semi-automated screening approach, yet were included in the meta-analyses in the included systematic reviews. [file 13643_2020_1528_MOESM4_ESM.docx]

**Additional file 4. Missed studies for systematic reviews with meta-analyses**

| **Systematic reviews with meta-analyses** | **Missed studies** |
| --- | --- |
| **Activity and pregnancy** | 1. Li HH et al. Reproductive health status of female workers in a railway system and possible influencing factors. Zhonghua Lao Dong Wei Sheng Zhi Ye Bing Za Zhi. 2017;35:566-70. (Chinese) |
| **Antipsychotics** | 1. Bobo MV et al. Antipsychotics and the risk of type 2 diabetes mellitus in children and youth. JAMA Psychiatry. 2013;70:1067-75. 2. Correll CU et al. Cardiometabolic risk of second-generation antipsychotic medications during first-time use in children and adolescents. JAMA 2009;302:1765-73. 3. McCracken JT et al. Risperidone in children with autism and serious behavioral problems. N Engl J Med. 2002;347:314-21. |
| **Brain injury** | None |
| **Digital technologies for pain** | None |
| **Treatments for bronchiolitis** | 1. Jartti et al. Short- and long-term efficacy of prednisolone for first acute rhinovirus-induced wheezing episode. J Allerg Clin Immunol. 2015;135:691-8. |
| **Visual acuity** | None |
