# Supplementary material for: Decoding semi-automated title-abstract screening: findings from a convenience sample of reviews
Source: Syst Rev. 2020 Nov 27;9:272. doi: 10.1186/s13643-020-01528-x (PMC7694314; doi:10.1186/s13643-020-01528-x)
Supplement: Supplementary file 5 — Additional file 5. Impact of the missed study on the results of relevant meta-analyses in the Antipsychotics systematic review. This table shows the pooled effect estimates for relevant meta-analyses with and without the study by McCracken et al. (2002), which was incorrectly classified as irrelevant via our semi-automated screening approach. [file 13643_2020_1528_MOESM5_ESM.docx]

**Additional file 5. Impact of the missed study on the results of relevant meta-analyses in the Antipsychotics systematic review** ^a^

| **Comparison** | **Original meta-analysis** | | | | **Meta-analyses without McCracken et al.** | | | |
| --- | --- | --- | --- | --- | --- | --- | --- | --- |
|  | **Trials** | **n** | **Pooled effect (95% CI)** | **p-value** | **Trials** | **n** | **Pooled effect (95% CI)** | **p-value** |
| **Autism spectrum disorder, second generation antipsychotic vs. placebo** | | | | | | | | |
| Irritability | 8 | 809 | MD -6.38 -8.68, -4.08) | <0.00001 | 7 | 708 | MD -5.47 (-7.29, -3.66) | <0.00001 |
| Lethargy/social withdrawal | 7 | 743 | MD -1.65 (-2.75, -0.56) | 0.003 | 6 | 642 | MD -1.41 (-2.58, -0.24) | 0.02 |
| Stereotypy | 7 | 743 | MD -1.73 (-2.83, -0.63) | 0.002 | 6 | 642 | MD -1.49 (-2.70, -0.27) | 0.02 |
| Inappropriate speech | 7 | 743 | MD -1.04 (-1.63, -0.45) | 0.0006 | 6 | 642 | MD -1.02 (-1.70, -0.34) | 0.003 |
| **Compulsions** | **5** | **568** | **MD -1.53 (-2.92, -0.15)** | **0.03** | **4** | **467** | **MD -1.17 (-2.70, 0.36)** | **0.14** |
| Response rate | 7 | 716 | RR 2.21 (1.51, 3.24) | <0.00001 | 6 | 615 | RR 1.89 (1.39, 2.58) | <0.00001 |
| Discontinuations (inefficacy) | 7 | 797 | RR 0.33 (0.13, 0.83) | 0.02 | 6 | 696 | RR 0.53 (0.33, 0.86) | 0.01 |
| Appetite increase | 7 | 599 | RR 2.38 (1.68, 3.38) | <0.00001 | 6 | 499 | RR 2.23 (1.29, 3.85) | 0.004 |

MD = mean difference; OR = odds ratio; RR = risk ratio.

^a^ Bolded rows
